# Upregulation of BMP1 through ncRNAs correlates with adverse outcomes and immune infiltration in clear cell renal cell carcinoma

**DOI:** 10.1186/s40001-023-01422-x

**Published:** 2023-10-17

**Authors:** Mancheng Gong, Shengxing Feng, Dongsheng Zhou, Jinquan Luo, Tianxin Lin, Shaopeng Qiu, Runqiang Yuan, Wenjing Dong

**Affiliations:** 1 Department of Urology, The People’s Hospital of Zhongshan, Zhongshan, 528403 Guangdong China; 2 Department of Urology, Sun Yat-sen Memorial Hospital, Guangzhou, 510080 Guangdong China; 3 Department of Urology, The First Affiliated Hospital of Sun Yat-sen University, Guangzhou, 510080 Guangdong China; 4 Department of Oncology, The People’s Hospital of Zhongshan, No. 2 Sunwen East Road, Zhongshan, 528403 Guangdong China

**Keywords:** BMP1, ccRCC, Prognosis, Survival, Immune cells

## Abstract

**Background:**

Renal cell carcinoma (RCC) accounts for approximately 2–3% of all adult malignancies. Clear cell renal cell carcinoma (ccRCC), which comprises 70–80% of all RCC cases, is the most common histological subtype.

**Methods:**

ccRCC transcriptome data and clinical information were downloaded from the TCGA database. We used the TCGA and GEPIA databases to analyze relative expression of BMP1 in various types of human cancer. GEPIA was used to perform survival analysis for BMP1 in various cancer types. Upstream binding miRNAs of BMP1 were obtained through several important target gene prediction tools. StarBase was used to predict candidate miRNAs that may bind to BMP1 and candidate lncRNAs that may bind to hsa-miR-532-3p. We analyzed the association between expression of BMP1 and immune cell infiltration levels in ccRCC using the TIMER website. The relationship between BMP1 expression levels and immune checkpoint expression levels was also investigated.

**Results:**

BMP1 was upregulated in GBM, HNSC, KIRC, KIRP and STAD and downregulated in KICH and PRAD. Combined with OS and DFS, BMP1 can be used as a biomarker for poor prognosis among patients with KIRC. Through expression analysis, survival analysis and correlation analysis, LINC00685, SLC16A1-AS1, PVT1, VPS9D1-AS1, SNHG15 and the CCDC18-AS1/hsa-miR-532-3p/BMP1 axis were established as the most potential upstream ncRNA-related pathways of BMP1 in ccRCC. Furthermore, we found that BMP1 levels correlated significantly positively with tumor immune cell infiltration, biomarkers of immune cells, and immune checkpoint expression.

**Conclusion:**

Our results demonstrate that ncRNA-mediated high expression of BMP1 is associated with poor prognosis and tumor immune infiltration in ccRCC.

## Introduction

Renal cell carcinoma (RCC) remains a major type of cancer, with a significant increase in incidence over the past years [1]. In 2019, there were approximately 73,750 new cases of kidney cancer in the United States, resulting in approximately 14,830 deaths [1]. Clear cell renal cell carcinoma (ccRCC) accounts for approximately 80% of clinical cases of renal cell carcinoma in adults [2]. Localized ccRCC can be treated by surgery and has good prognosis, but approximately 20% of cases are at an advanced stage at the time of diagnosis, resulting in relatively poor prognosis [1, 3]. Despite current application of tyrosine kinase inhibitor (TKI) therapy and immunotherapy, the median survival time for advanced ccRCC is only approximately 3–4 years [4–6]. Moreover, TKI is associated with increased risk of multiple cardiovascular events, and immunotherapy may increase the occurrence of such adverse reactions [7]. Therefore, it is very important to understand the mechanism of development and progression of ccRCC.

Bone morphogenic proteins (BMPs) participate in cartilage and bone formation. BMP1 is a metalloendopeptidase belonging to the astacin superfamily and is a splice variant of mammalian tolloid protein, whereas BMP2 to BMP16 belong to the transforming growth factor-β (TGF-β) superfamily [8]. BMP is commonly found in various tissues of the human body. BMPs participate in the genesis and development of tumors by inducing apoptosis and inhibiting proliferation through regulation of the epithelial–mesenchymal transition (EMT), the G2M checkpoint, angiogenesis, and the hypoxia pathway [9, 10]. BMP1 can activate the TGF-β signaling pathway, which initiates cleavage and release of the TGF-β complex from the extracellular matrix [11]. In recent years, it was found that BMP1 is highly expressed in some cancers and associated with cancer invasiveness in gastric cancer [12], lung cancer [13], osteosarcoma [14], colon cancer [15] and renal cancer [10]. However, expression and the mechanism of BMP1 in ccRCC and how they relate to prognosis are still unresolved. In addition, current understanding regarding the association between BMP1 and tumor immune infiltration in ccRCC remains unclear.

To address these questions, we first investigated expression of BMP1 and its prognostic significance in a variety of human cancers. Then, the mechanism of noncoding RNA (ncRNA)-associated regulation of BMP1 in ccRCC was examined. Finally, we explored the relationship of BMP1 expression with biomarkers of immune cells, immune cell infiltration, and immune checkpoints in ccRCC. Overall, our results reveal that high expression of BMP1 in ccRCC is associated with poor prognosis and tumor immune infiltration through ncRNAs.

## Materials and methods

### Expression of BMP1 in 18 cancer types

The TCGA database, including clinical data, miRNA expression, mRNA expression, genome variation and methylation data of various human cancers, is a very useful cancer database [16]. In this study, we analyzed expression of BMP1 in 18 cancer types (BRCA, CHOL, BLCA, COAD, ESCA, HNSC, GBM, KICH, KIRP, KIRC, LUAD, LIHC, LUSC, PRAD, READ, THCA, STAD, and UCEC) using a Mann–Whitney *U* test with the R package ggplot2.

### GEPIA database analysis

GEPIA (http://gepia.cancer-pku.cn/), a web tool including TCGA and Genotype-Tissue Expression (GTEx) data, was adopted to research BMP1 expression in various types of human cancer [16]. GEPIA was used to perform survival analysis, including OS and DFS, for BMP1 in 18 various cancer types. The survival R package was used to determine the prognostic value of candidate lncRNAs in ccRCC.

### Detected upstream binding miRNAs of BMP1

Through several important target gene prediction tools, including PicTar, PITA, RNA22, miRmap, miRanda, microT and TargetScan, upstream binding miRNAs of BMP1 were obtained. These obtained miRNAs were deemed candidate miRNAs of BMP1.

### StarBase database analysis

StarBase (http://starbase.sysu.edu.cn/) is an important database for performing miRNA-related research [17]. StarBase was used to predict candidate miRNAs that may bind to BMP1 and candidate lncRNAs that may bind to hsa-miR-532-3p.

### Analysis of the potential association between BMP1 expression and immune-related factors

TIMER (https://cistrome.shinyapps.io/timer/) is a very important website that is often used to analyze tumor-infiltrating immune cells [18]. We analyzed the association of expression of BMP1 and immune cell infiltration levels using the TIMER website in ccRCC. The relationship between BMP1 expression levels and immune checkpoint expression levels was also investigated and visualized using Spearman’s rho value within TIMER.

### Statistical analysis

Statistical analysis was automatically calculated using either the aforementioned online database or R software (v.4.1.3). Statistical significance was determined when the p-value or the log-rank p-value was less than 0.05.

## Results

### Analysis of BMP1 expression across cancers

**Fig. 1:**
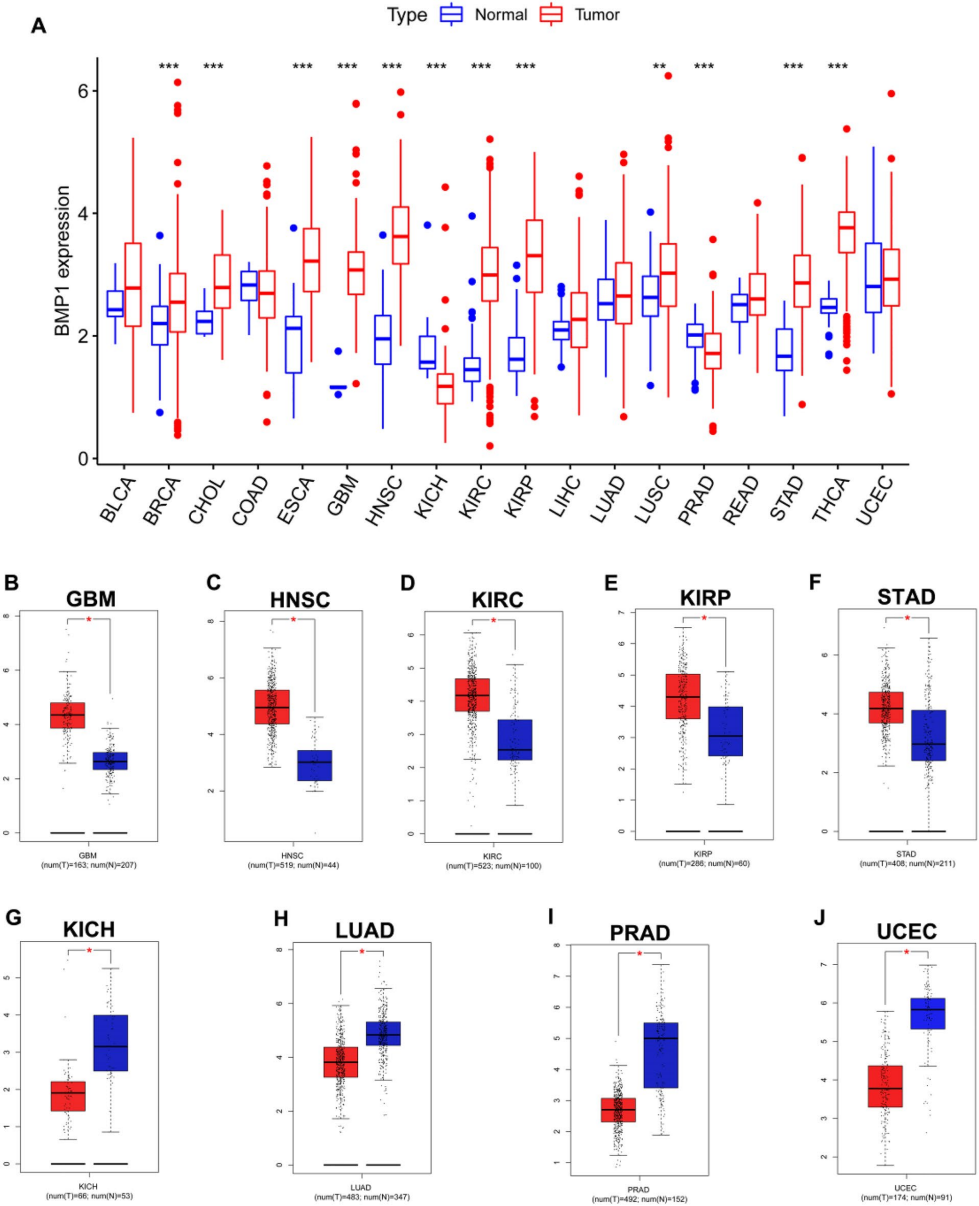
Detection of BMP1 expression in multiple cancers. **A** BMP1 expression in 18 types of human cancer based on TCGA. **B**–**J** Expression of BMP1 in TCGA cancer tissues compared with corresponding TCGA and GTEx normal tissues. *p-value < 0.05; **p-value < 0.01; ***p-value < 0.001

We first detected expression of BMP1 in 18 types of human cancer to illuminate the roles of BMP1 in carcinogenesis using the TCGA database. We found that BMP1 was highly expressed in 10 cancer types compared to normal samples, including BRCA, CHOL, GBM, HNSC, ESCA, KIRC, KIRP, LUSC, STAD and THCA, but was expressed at low levels in 2 cancer types, KICH and PRAD. However, no significant difference in BMP1 in BLCA, COAD, LIHC, LUAD, READ and UCEC was detected (Fig. 1A). Then, we further examined BMP1 expression in these 18 cancer types through the GEPIA database. As shown in Fig. 1B–F, expression of BMP1 in GBM, HNSC, KIRC, KIRP and STAD was markedly upregulated compared with that in corresponding normal controls; in contrast, it was obviously downregulated in KICH, LUAD, PRAD and UCEC (Fig. 1G–J). Overall, BMP1 was upregulated in GBM, HNSC, KIRC, KIRP and STAD and downregulated in KICH and PRAD. These results suggest that BMP1 may play an important role in the carcinogenesis of these 7 cancers.

### Relationship between BMP1 and prognosis of human cancer

**Fig. 2:**
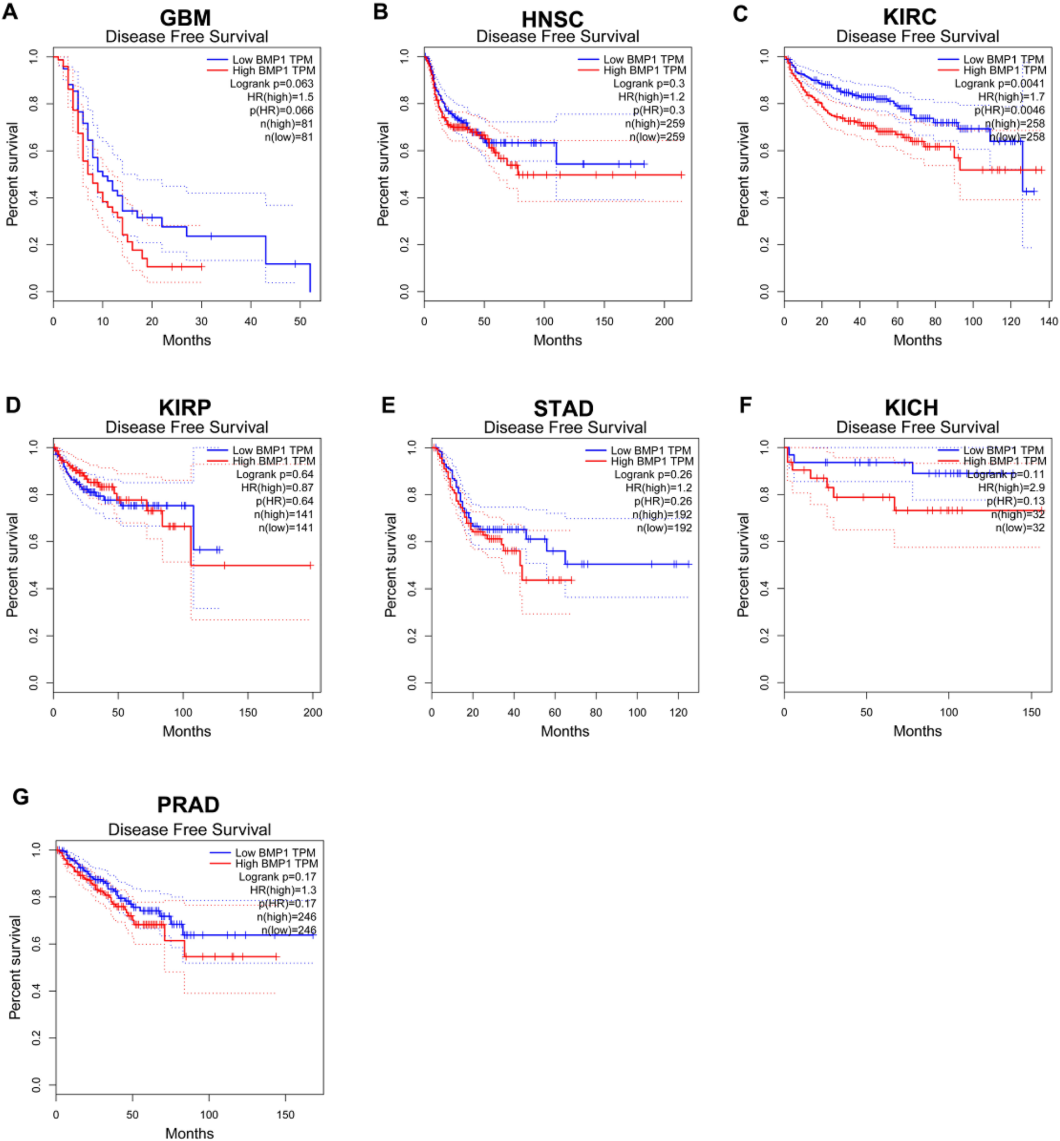
Overall survival (OS) in relation to BMP1 in various human cancers was analyzed using the GEPIA database. **A**–**G** The OS plot for BMP1 in GBM (**A**), HNSC (**B**), KIRC (**C**), KIRP (**D**), STAD (**E**), KICH (**F**) and PRAD (**G**)

**Fig. 3:**
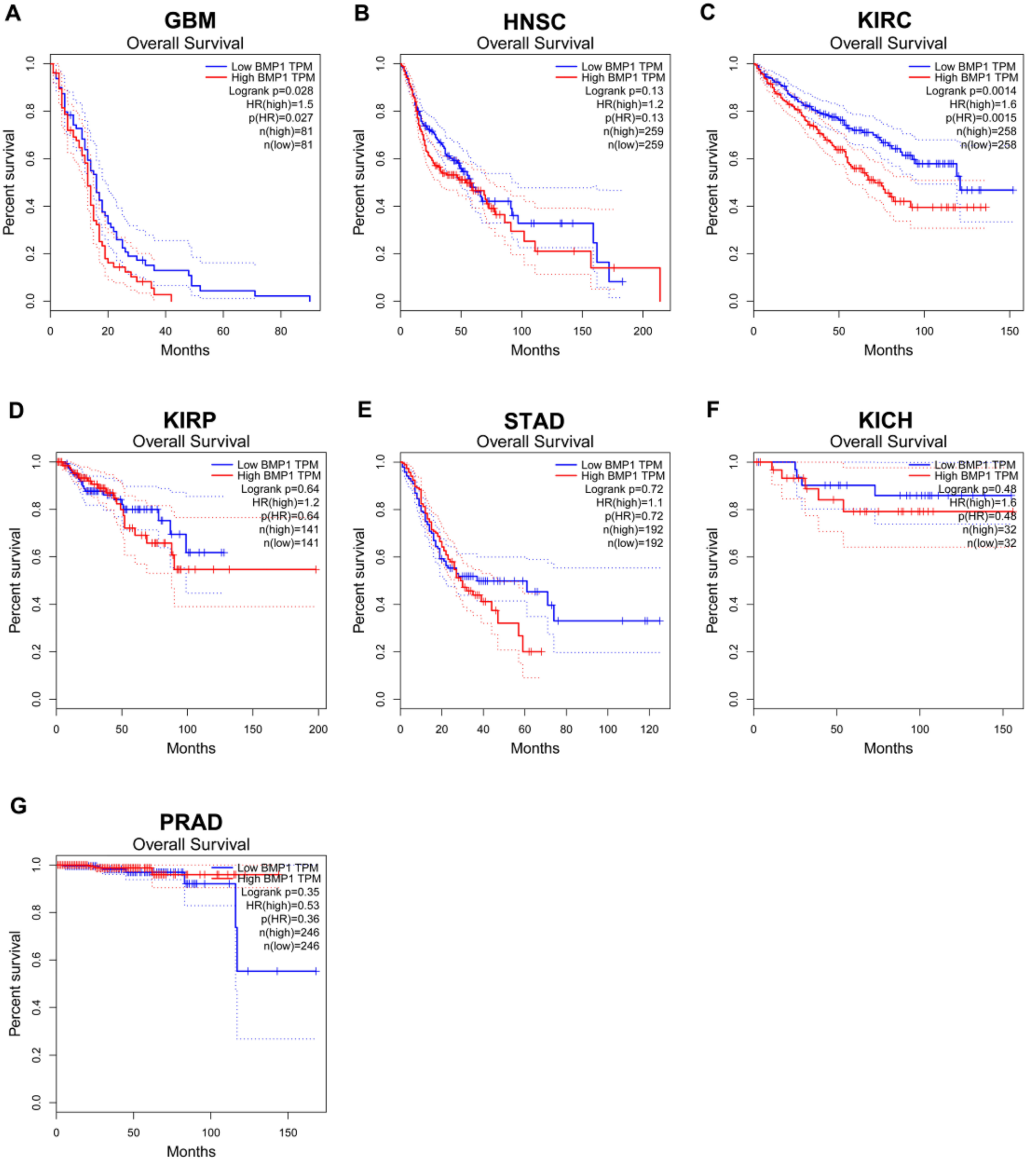
Disease-free survival (DFS) in relation to BMP1 in various human cancers was determined using the GEPIA database. **A**–**G** The RFS plot for BMP1 in GBM (**A**), HNSC (**B**), KIRC (**C**), KIRP (**D**), STAD (**E**), KICH (**F**) and PRAD (**G**)

Next, we conducted survival analysis, including disease-free survival (DFS) and overall survival (OS), for BMP1 in GBM, HNSC, KIRC, KIRP, STAD, KICH and PRAD. For OS, high expression of BMP1 in GBM (log-rank *p* = 0028, HR = 1.5, *p* = 0.027) and KIRC (log-rank *p* = 0.0014, HR = 1.6, *p* = 0.0015) was associated with poor prognosis (Fig. 2). For DFS, increased expression of BMP1 indicated unfavorable prognosis in KIRC (log-rank *p* = 0.0041, HR = 1.7, *p* = 0.0046) among all cancer types (Fig. 3). BMP1 was not statistically significant in predicting the prognosis of patients with other types of cancer. Combined with OS and DFS, BMP1 can be used as a biomarker for poor prognosis in patients with KIRC.

### Prediction and analysis of upstream miRNAs of BMP1

**Fig. 4:**
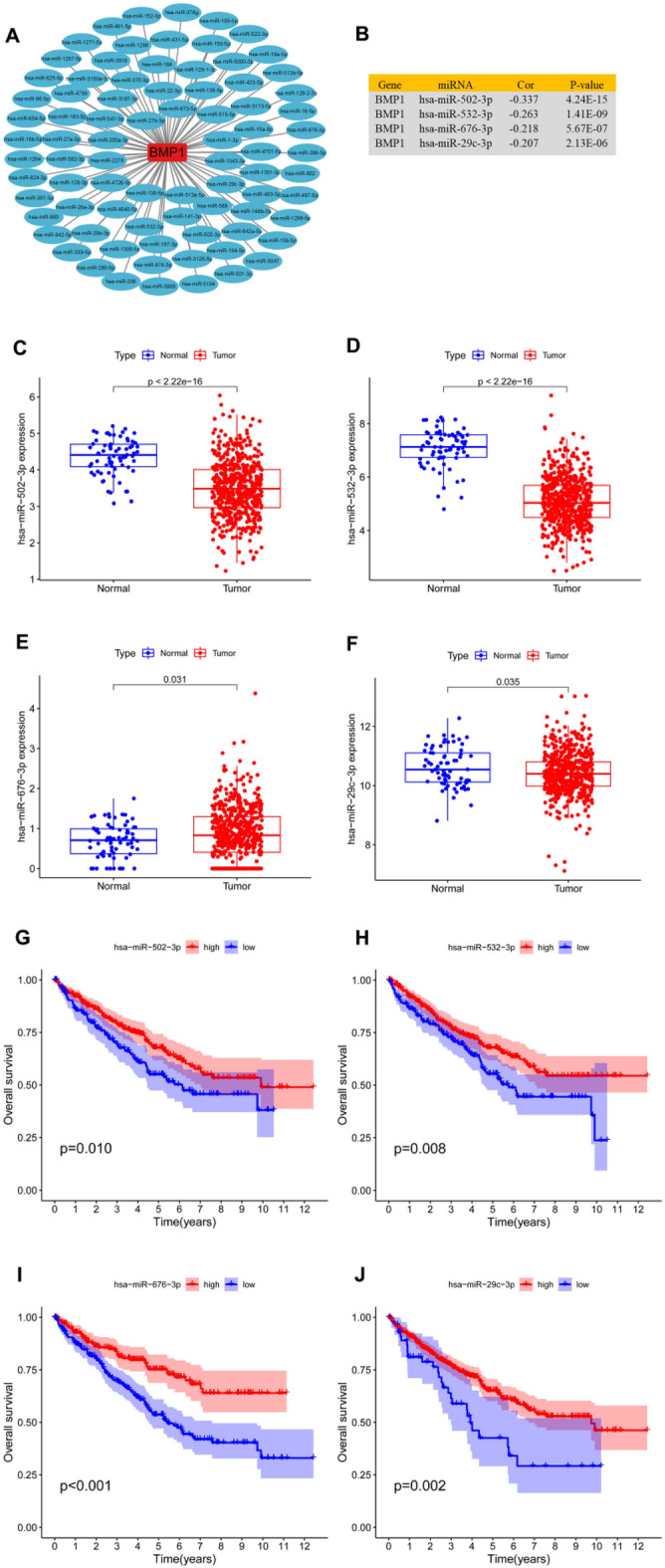
Investigation of upstream miRNAs of BMP1 in ccRCC. **A** The miRNA-BMP1 regulatory network established using Cytoscape software. **B** Correlation between predicted miRNAs and BMP1 expression in ccRCC analyzed using the starBase database. **C**–**F** Expression of hsa-miR-502-3p, hsa-miR-532-3p, hsa-miR-676-3p and hsa-miR-29c-3p in ccRCC and control normal samples assessed by the starBase database. **G**–**J** The prognostic value of hsa-miR-502-3p, hsa-miR-532-3p, hsa-miR-676-3p and hsa-miR-29c-3p in ccRCC determined by Kaplan‒Meier plotter

The regulatory function of ncRNAs on gene expression has been explored in depth. To detect whether BMP1 is regulated by ncRNAs, we first explored upstream miRNAs that may bind to BMP1 through several target gene prediction tools, including PITA, RNA22, PicTar, miRmap, microT, miRanda and TargetScan (Fig. 4A). Through coexpression analysis with BMP1, 4 miRNAs were selected (Fig. 4B). The expression and prognostic value of hsa-miR-676-3p, hsa-miR-502-3p, hsa-miR-532-3p and hsa-miR-29c-3p in ccRCC were investigated. The results (Fig. 4C–J) showed that hsa-miR-502-3p, hsa-miR-532-3p and hsa-miR-29c-3p were all significantly downregulated in ccRCC and that their downregulation was positively linked to patient prognosis. We found that hsa-miR-676-3p was upregulated in ccRCC and that patients with high expression of hsa-miR-676-3p had better prognosis. As miRNA and BMP1 should correlate negatively based on the mechanism by which miRNAs regulate target gene expression, we finally chose hsa-miR-532-3p for further analysis.

### Prediction and analysis of upstream lncRNAs of hsa-miR-532-3p

**Fig. 5:**
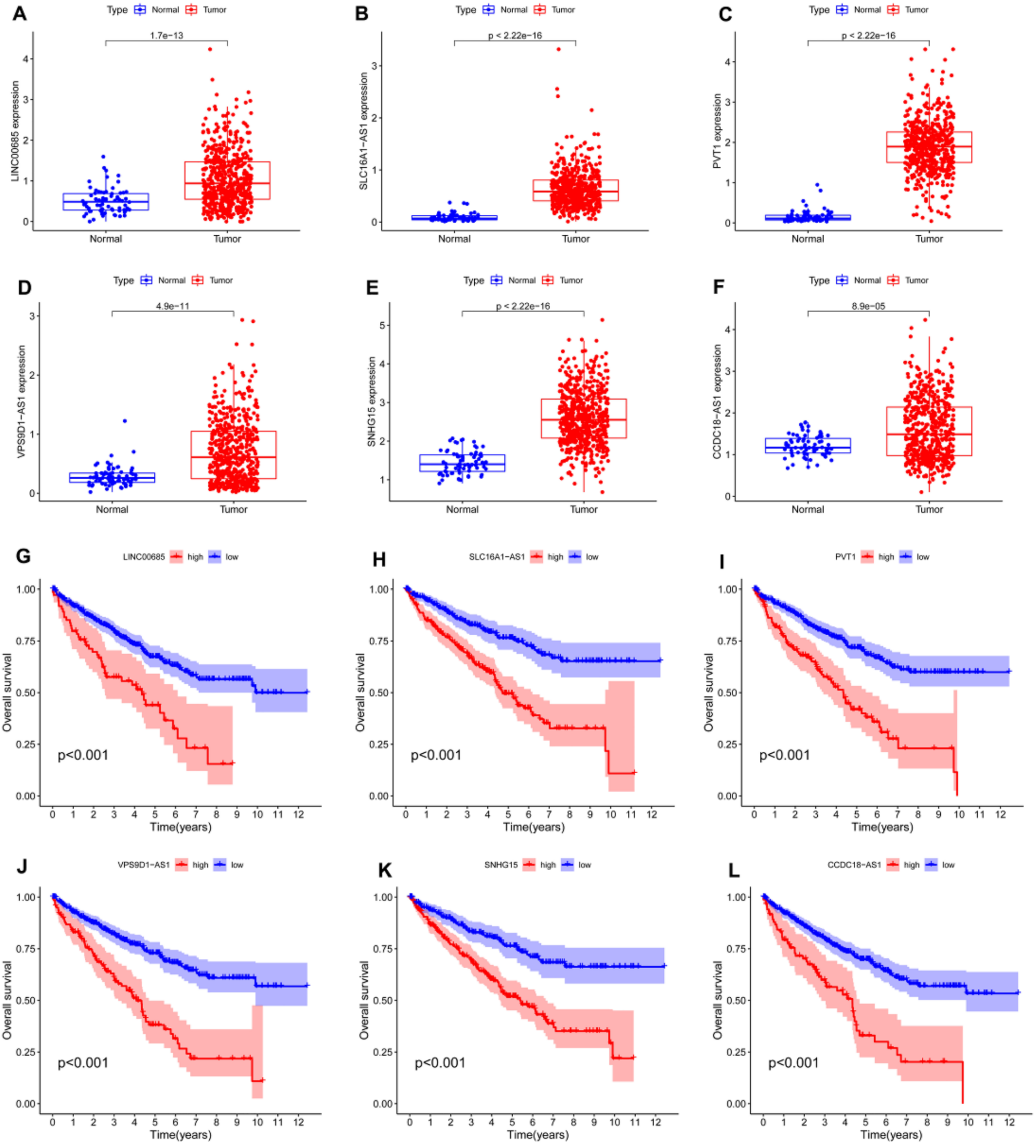
Expression and relation to prognosis of upstream lncRNAs of hsa-miR-532-3p were analyzed in ccRCC. **A**–**F** Expression of LINC00685 (**A**), SLC16A1-AS1 (**B**), PVT1 (**C**), VPS9D1-AS1 (**D**), SNHG15 (**E**) and CCDC18-AS1 (**F**) in TCGA ccRCC compared with “TCGA normal” or “TCGA and GTEx normal” data. **G**–**L** OS analysis for LINC00685 (**G**), SLC16A1-AS1 (**H**), PVT1 (**I**), VPS9D1-AS1 (**J**), SNHG15 (**K**) and CCDC18-AS1 (**L**) in ccRCC

**Fig. 6:**
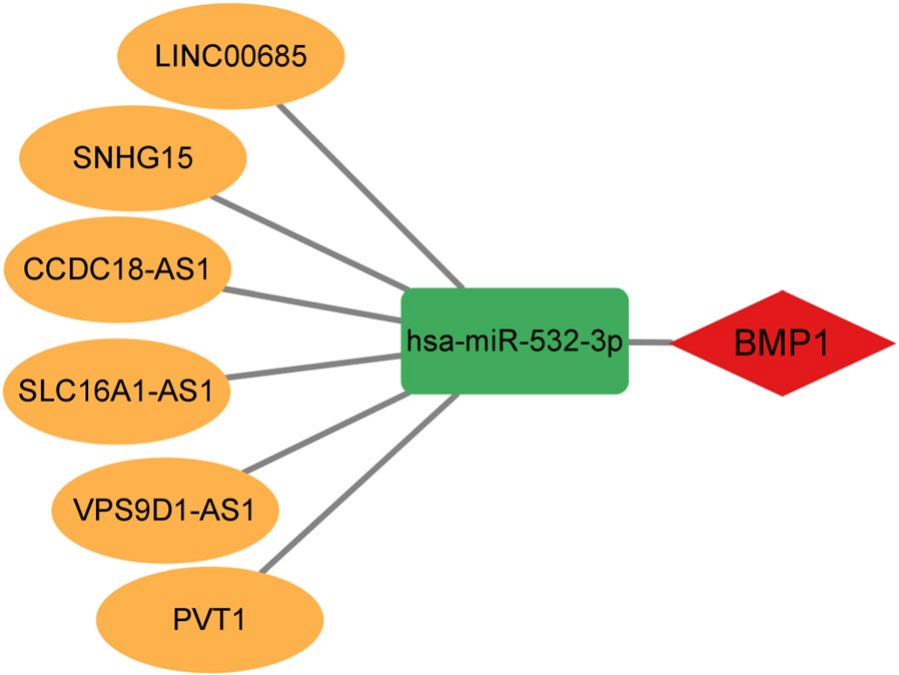
The model of LINC00685, SLC16A1-AS1, PVT1, VPS9D1-AS1, SNHG15 and the CCDC18-AS1/hsa-miR-532-3p/BMP1 axis in ccRCC carcinogenesis

We predicted upstream lncRNAs of hsa-miR-532-3p using the starBase database, and 148 possible lncRNAs were obtained. According to the competing endogenous RNA (ceRNA) hypothesis, lncRNAs can competitively bind shared miRNAs to increase mRNA expression. Hence, there should be a negative correlation between lncRNAs and miRNAs or a positive correlation between lncRNAs and mRNAs. In total, 6 lncRNAs were selected for further analysis (Table 1). LINC00685, SLC16A1-AS1, PVT1, VPS9D1-AS1, SNHG15 and CCDC18-AS1 were significantly upregulated in ccRCC compared with normal controls (Fig. 5A–F). Subsequently, the prognostic values of the six lncRNAs in ccRCC were investigated. As shown in Fig. 5G–L, ccRCC patients with high expression of these six lncRNAs had poor prognosis. To improve visualization, a lncRNA‒miRNA-BMP1 regulatory network was constructed using Cytoscape software (Fig. 6).

**Table 1:** Correlation analysis between lncRNAs and hsa-miR-532-3p in ccRCC determined by the starBase database

lncRNA	miRNA	Cor	*P*-value
LINC00685	hsa-miR-532-3p	− 0.155	3.90E-04
SLC16A1-AS1	hsa-miR-532-3p	− 0.182	3.31E-05
PVT1	hsa-miR-532-3p	− 0.159	2.86E-04
VPS9D1-AS1	hsa-miR-532-3p	− 0.145	9.42E-04
SNHG15	hsa-miR-532-3p	− 0.176	5.73E-05
CCDC18-AS1	hsa-miR-532-3p	− 0.149	6.65E-04

### Relationships between BMP1 and immune cell infiltration in ccRCC

**Fig. 7:**
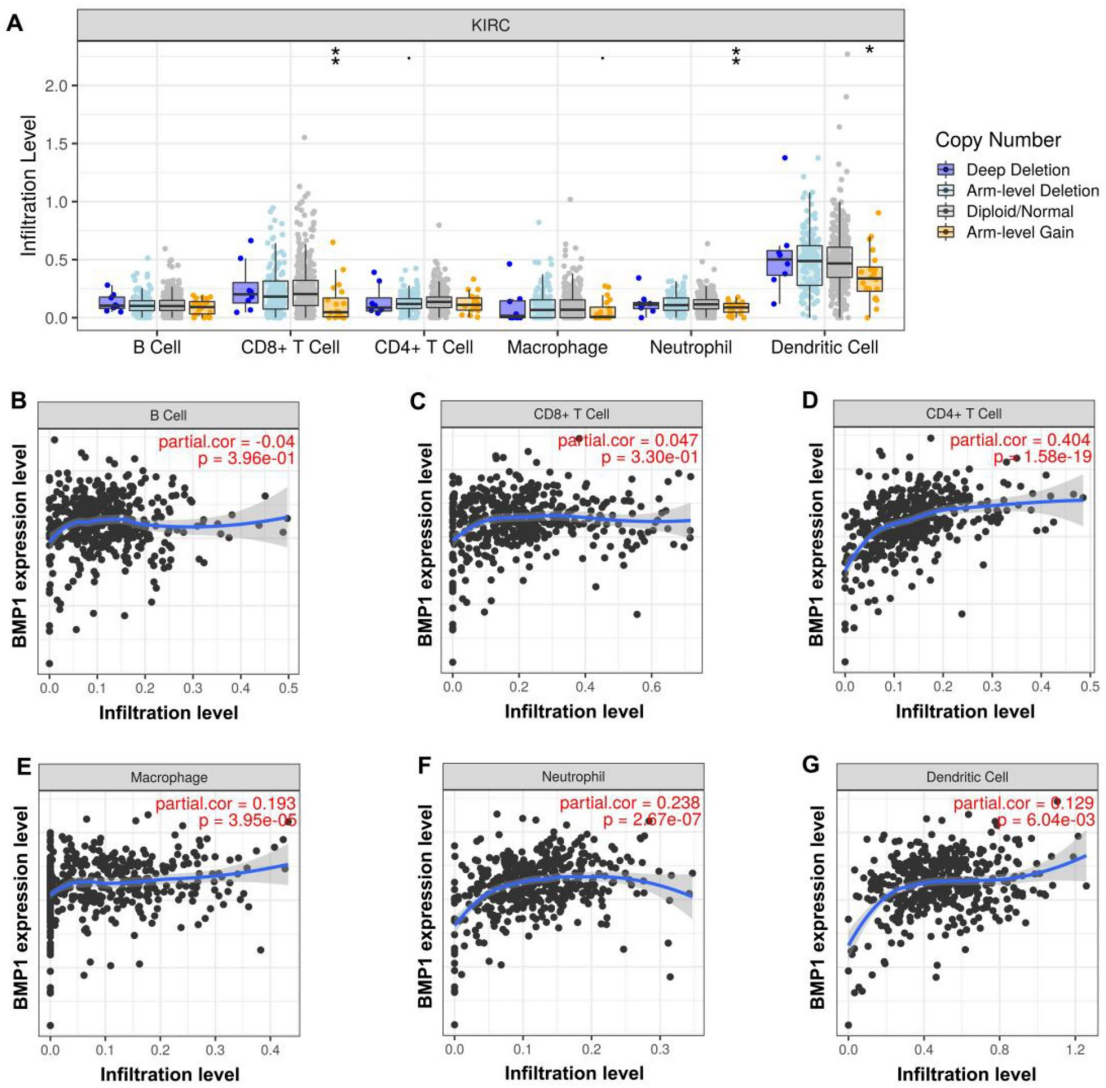
The relationship between immune cell infiltration and BMP1 expression level in ccRCC. **A** Different infiltration levels of immune cells under different BMP1 copy numbers in ccRCC. **B**–**G** The relationship of BMP1 expression level with B-cell (**B**), CD8^+^ T-cell (**C**), CD4^+^ T-cell (**D**), macrophage (**E**), neutrophil (**F**), or dendritic cell (**G**) infiltration level in ccRCC

We explored relationships between BMP1 and immune cell infiltration in ccRCC using TIMER. The results showed that the immune cell infiltration levels of CD8^+^ T cells, neutrophils, and dendritic cells correlated with the copy numbers of BMP1 in ccRCC (Fig. 7A). However, no significant change in the immune cell infiltration level of B cells, CD4^+^ T cells, or macrophages under various copy numbers of BMP1 in ccRCC was observed (Fig. 7A). Then, we investigated the correlation between the immune cell infiltration level and BMP1 expression level. As shown in Fig. 7B–G, BMP1 expression was obviously positively associated with all analyzed immune cells, including B cells, CD8^+^ T cells, CD4^+^ T cells, macrophages, neutrophils, and dendritic cells, in ccRCC.

### Relationships between BMP1 and biomarkers of immune cells in ccRCC

To further study the role of BMP1 in tumor immunity, we investigated relationships between expression of BMP1 and biomarkers of immune cells in ccRCC. As presented in Table 2, Expression of BMP1 correlated significantly positively with B-cell biomarkers (CD19 and CD79A), CD4^+^ T-cellcell biomarkers (CD4), CD8^+^ T-cell biomarkers (CD8B), neutrophil biomarkers (ITGAM and CCR7), M1 macrophage biomarkers (NOS2, PTGS2, and IRF5), M2 macrophage biomarkers (CD163, VSIG4, and MS4A4A) and dendritic cell biomarkers (HLA-DPB1, CD1C, NRP1, and ITGAX) in ccRCC.

**Table 2:** Correlation analysis between BMP1 and biomarkers of immune cells in ccRCC determined by the GEPIA database

Immune cell	Biomarker	Cor	*P*-value
B-cell	CD19	0.311	2.61E-13
	CD79A	0.230	7.63E-08
CD8 + T-cell	CD8A	0.083	5.38E-02
	CD8B	0.090	3.82E-02
CD4 + T-cell	CD4	0.232	5.96E-08
M1 macrophage	NOS2	0.159	2.28E-04
	IRF5	0.152	4.31E-04
	PTGS2	0.153	3.90E-04
M2 macrophage	CD163	0.148	6.07E-04
	VSIG4	0.275	1.09E-10
	MS4A4A	0.157	2.65E-04
Neutrophil	CEACAM8	0.027	5.30E-01
	ITGAM	0.208	1.29E-06
	CCR7	0.252	3.73E-09
Dendritic cell	HLA-DPB1	0.092	3.31E-02
	HLA-DQB1	0.032	4.60E-01
	HLA-DRA	0.012	7.79E-01
	HLA-DPA1	0.028	5.23E-01
	CD1C	0.096	2.64E-02
	NRP1	0.135	1.72E-03
	ITGAX	0.252	4.13E-09

### Correlation of BMP1 expression with immune checkpoints in ccRCC

**Fig. 8:**
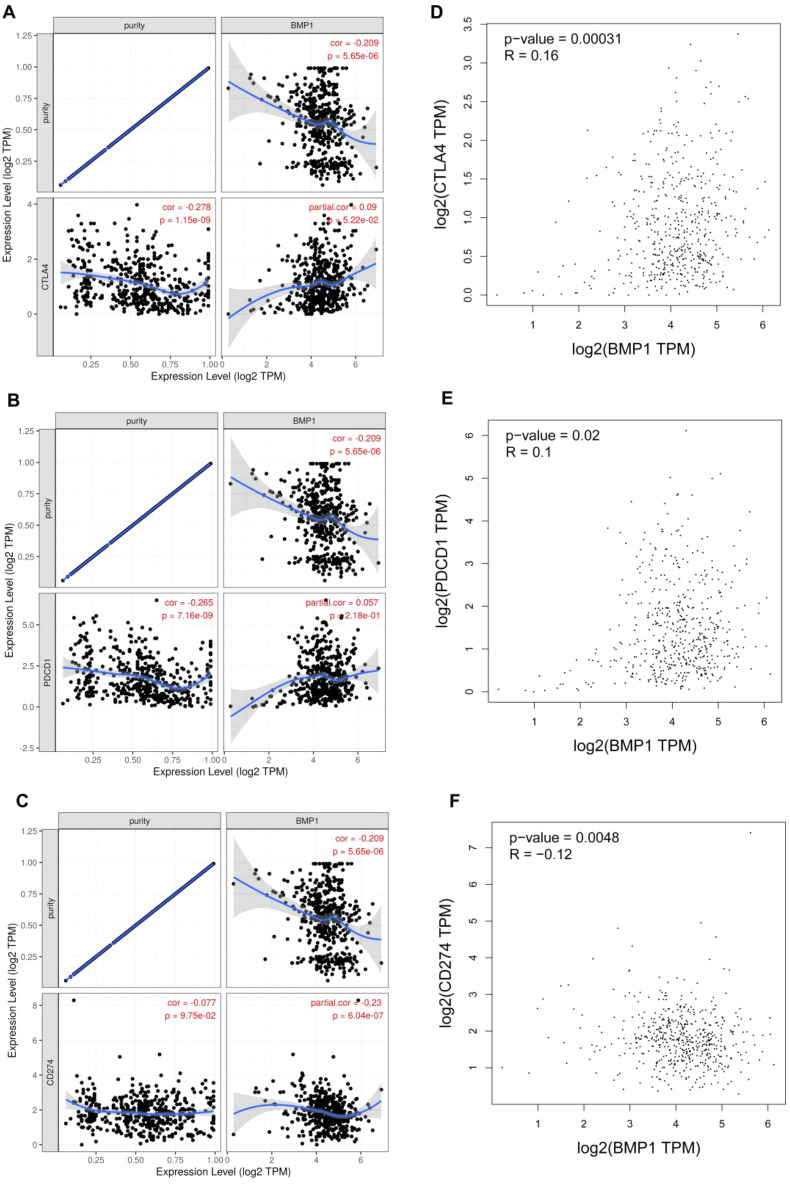
The relationship of BMP1 expression with PD-1, PD-L1, and CTLA-4 expression levels in ccRCC. **A** The relationship between BMP1 and the expression of PD-1 in ccRCC determined by purity using TIMER. **B** Correlation of BMP1 with expression of PD-L1 in ccRCC determined by purity using TIMER. **C** The relationship of SEMA3F with expression of CTLA-4 in ccRCC determined by purity using TIMER. **D** The expression correlation of BMP1 with PD1 in ccRCC using the GEPIA database. **E** The expression correlation of BMP1 with PD-L1 in ccRCC using the GEPIA database. **F** The expression correlation of BMP1 with CTLA-4 in ccRCC using the GEPIA database

CTLA-4 and PD1/PD-L1 are important immune checkpoints that are related to the effect of tumor immunotherapy. Considering the potential oncogenic role of BMP1 in ccRCC, we evaluated the association of BMP1 with CTLA-4, PD1 or PD-L1. Based on TIMER, expression of BMP1 correlated significantly positively with CTLA-4 and PD1 in ccRCC, which was adjusted by purity. However, expression of BMP1 correlated significantly negatively with that of PD-L1 in ccRCC (Fig. 8A–C). Through the GEPIA database, we also found that BMP1 correlated significantly positively with CTLA-4 and PD1 in ccRCC but that BMP1 correlated significantly negatively with PD-L1 (Fig. 8D–F). These results suggest that BMP1 is related to the effect of immunotherapy on ccRCC.

## Discussion

In this study, we identified BMP1 as being upregulated in GBM, HNSC, KIRC, KIRP, and STAD. Additionally, we found that BMP1 can serve as a biomarker for poor prognosis in patients with KIRC. We also established the LINC00685, SLC16A1-AS1, PVT1, VPS9D1-AS1, SNHG15, and CCDC18-AS1/hsa-miR-532-3p/BMP1 axis as the most potential upstream ncRNA-related pathway of BMP1 in ccRCC. Moreover, our findings revealed a significant positive correlation between BMP1 levels and tumor immune cell infiltration, biomarkers of immune cells, and immune checkpoint expression.

We first investigated expression of BMP1 across cancers using TCGA data, after which the GEPIA database was also used to confirm BMP1 expression. Survival analysis for BMP1 in cancer types of interest indicated that ccRCC patients with high expression of BMP1 had poor prognosis. The preliminary results of Xiao et al. [10] also demonstrated that overexpression of BMP1 is related to short survival time in ccRCC patients. The results of this report and our analysis demonstrate the carcinogenic role of BMP1 in ccRCC.

LncRNAs sponge miRNAs and regulate miRNA-targeted mRNAs at the posttranscriptional level in the cytoplasm [19, 20]. To explore the ceRNA mechanism of BMP1, we used 7 prediction programs to find possible miRNAs that might bind to BMP1, and 4 miRNAs were finally obtained. Survival analysis showed that ccRCC patients with high expression of these 4 miRNAs had better prognosis. Research has shown that miRNAs interact with the 3’-UTR of target genes to decrease the protein level by impeding the translation process or enhancing degradation of respective mRNAs [21]. For instance, hsa-miR-532-3p acts as a tumor suppressor and inhibits cell proliferation in lung cancer, breast cancer, ovarian cancer and renal cell carcinoma by targeting ETS1 [22, 23]. Hsa-miR-29c-3p modulates FOS expression to repress EMT and cell proliferation in age-related cataract tissues [24]. Combined with the results of survival analysis and differential expression of miRNAs in renal cancer and normal renal tissues, we selected hsa-miR-532-3p for further analysis.

Based on the ceRNA hypothesis [25], the potential lncRNAs that regulate hsa-miR-532-3p should be oncogenic in ccRCC. Next, upstream lncRNAs of the hsa-miR-532-3p/BMP1 axis were predicted, and 148 potential lncRNAs were identified. Combined with expression analysis, correlation analysis and survival analysis, six of the most likely upregulated lncRNAs, including LINC00685, SLC16A1-AS1, PVT1, VPS9D1-AS1, SNHG15 and CCDC18-AS1, were obtained. It has been reported that most of these six lncRNAs play a role as oncogenes in a variety of malignant tumors, including ccRCC. For example, SNHG15 stimulates renal cell carcinoma proliferation and EMT by regulating the NF-κB signaling pathway [26]. Downregulation of SLC16A1-AS1 inhibits the proliferation, viability and migration of ccRCC [27]. PVT1 is significantly upregulated in ccRCC tissues, and high expression of PVT1 is associated with poor prognosis in ccRCC patients [28, 29]. In general, LINC00685, SLC16A1-AS1, PVT1, VPS9D1-AS1, SNHG15 and the CCDC18-AS1/hsa-miR-532-3p/BMP1 axis were deemed to be potential regulatory pathways in ccRCC.

In recent years, the tumor microenvironment (TME) has gained much attention and is considered to be a key factor affecting treatment resistance, tumor development and prognosis [30–32]. Our results demonstrate that BMP1 is significantly positively associated with various immune cells, including CD4^+^ T cells, B cells, CD8^+^ T cells, macrophages, neutrophils, and dendritic cells, in ccRCC. Furthermore, BMP1 is significantly positively associated with biomarkers of these infiltrated immune cells. These findings indicate that BMP1 might regulate development of ccRCC through tumor immune infiltration. In addition, the effectiveness of immunotherapy depends not only on adequate infiltration of immune cells into the tumor microenvironment but also on adequate expression of immune checkpoints [33]. Thus, we also investigated the relationship between BMP1 and immune checkpoints. Our results suggest that BMP1 correlates significantly positively with CTLA-4 and PD1 but significantly negatively with PD-L1 in ccRCC. These results indicate that targeting BMP1 might increase the efficacy of immunotherapy in ccRCC.

In general, we demonstrate that BMP1 is highly expressed in multiple types of human cancer (including ccRCC) and strongly correlates with unfavorable prognosis in ccRCC. We explored the upstream regulatory mechanism of BMP1 in ccRCC, namely, LINC00685, SLC16A1-AS1, PVT1, VPS9D1-AS1, SNHG15 and the CCDC18-AS1/hsa-miR-532-3p/BMP1 axis (Fig. 6). In addition, our results suggest that BMP1 might exert its oncogenic roles by increasing tumor immune cell infiltration and immune checkpoint expression.

Although our study has some advantages, it also has some limitations. First, the results were obtained from the TCGA database only, and verification in other databases is needed. Second, all the results were obtained by analyzing public datasets. In addition, further experiments should be performed in vivo and in vitro to verify these results.

## Data Availability

The datasets generated and analyzed during the current study are publicly available in TCGA (https://portal.gdc.cancer.gov) and GEPIA (http://gepia.cancer-pku.cn/).

## Abbreviations

RCC: Renal cell carcinoma
ccRCC: Clear cell renal cell carcinoma
BMP: Bone morphogenic protein
BRCA: Breast cancer
BLCA: Bladder urothelial carcinoma
CHOL: Cholangiocarcinoma
COAD: Colon adenocarcinoma
ESCA: Esophageal carcinoma
HNSC: Head and neck squamous cell carcinoma
GBM: Glioblastoma multiforme
KICH: Kidney chromophobe
KIRP: Kidney renal papillary cell carcinoma
KIRC: Kidney renal clear cell carcinoma
LUAD: Lung adenocarcinoma
LIHC: Liver hepatocellular carcinoma
LUSC: Lung squamous cell carcinoma
PRAD: Prostate adenocarcinoma
READ: Rectum adenocarcinoma
THCA: Thyroid carcinoma
STAD: Stomach adenocarcinoma
UCEC: Uterine corpus endometrial carcinoma
